# A rare case of isolated persistent left superior vena cava diagnosed by echocardiography

**DOI:** 10.1186/s13019-024-02709-8

**Published:** 2024-04-04

**Authors:** Dorota Smolarek, Hanna Jankowska, Karolina Dorniak, Marcin Hellmann

**Affiliations:** Department of Cardiac Diagnostics, Medical University of Gdansk, Smoluchowskiego 17, Gdansk, 80-214 Poland

**Keywords:** Persistent left superior vena cava, Coronary sinus, Echocardiography

## Abstract

**Background:**

The persistent left superior vena cava (PLSVC) is an infrequent vascular variant. PLSVC with absent right superior vena cava, also known as isolated PLSVC, is an exceptionally rare entity. In this case we present a patient with isolated PLSVC draining to coronary sinus, diagnosed incidentally during echocardiography.

**Case presentation:**

A 35-year-old man underwent a transthoracic echocardiography which showed an enormously dilated coronary sinus. Hand-agitated saline was injected via peripheral intravenous cannulas. The contrast appeared firstly in the coronary sinus before it opacified the right atrium. Since this was also visible by the right antecubital saline injection, it indicated an extremely rare case of PLSVC with the absence of right superior vena cava which was confirmed by cardiac magnetic resonance.

**Conclusions:**

The finding of a distinctively dilated coronary sinus in echocardiography led us to further investigation using agitated saline that revealed an infrequent anomaly termed isolated PLSVC. The in-depth diagnosis of this vascular variant is crucial considering that it may lead to important clinical implications, such as difficulties with central venous access, especially in the current era of a rapid development of cardiac device therapies.

**Supplementary Information:**

The online version contains supplementary material available at 10.1186/s13019-024-02709-8.

## Background

The persistent left superior vena cava (PLSVC) is a remnant of the embryonic left anterior cardinal vein [1]. Most cases of PLSVCs coexist with the presence of right-sided vein and this condition is recognised as superior vena cava (SVC) duplication. PLSVC with the agenesis of right SVC, also known as isolated PLSVC, is an exceptionally rare entity [2]. Almost half of patients with this vascular variant have additional cardiac anomalies such as atrial septal defect, endocardial cushion defects, or tetralogy of Fallot [3].

## Case presentation

**Fig. 1:**
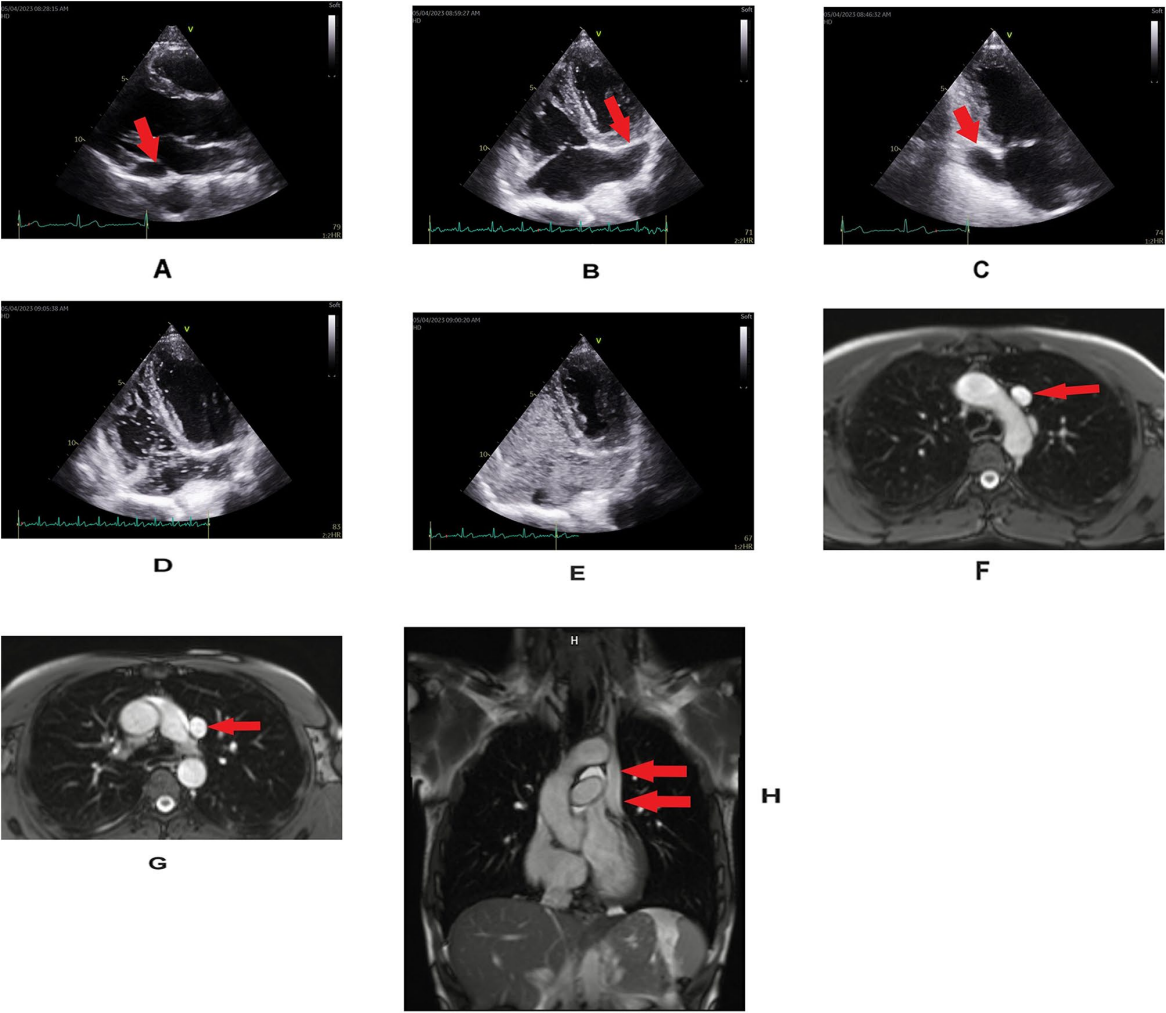
**A**, **B**, **C**, **D**, **E**. Transthoracic echocardiography. **A**, **B**, **C**. Significantly dilated coronary sinus (arrows)- dimensions; parasternal long axis view- 2.3 × 1.4 cm (**A**), four-chamber view- 2.2 cm (**B**), two-chamber view- 2.9 × 1.7 cm (**C**). **D**, **E**. The injection of agitated saline into the right arm vein resulting in the opacification of a dilated coronary sinus followed by right atrial filling. **F**, **G**, **H**. CMR. Single-shot SSFP images, showing a single persistent left SVC (arrows), with the right-sided SVC missing in its typical position to the right and posteriorly from ascending aorta. **F**, **G**. The image from an axial stack. **H**. The coronal image CMR-cardiac magnetic resonance, SSFP-steady state free precession sequence, SVC-superior vena cava

A 35-year-old man with systemic sclerosis (SSc) was referred to our cardiology outpatient clinic for a routine transthoracic echocardiography. The diagnosis of SSc was made four years earlier on the basis of a clinical picture and presence of autoantibodies (anti-Scl-70). The main symptoms regarded a skin thickening of the fingers of both hands, fingertip lesions, darker skin pigmentation and microcheilia. Moreover, the features of interstitial lung disease were identified in computed tomography and ultrasonography. No cardiovascular complaints were present. Transthoracic echocardiography showed a normal size of ventricles with preserved systolic function, low probability of pulmonary hypertension and enormously dilated coronary sinus (CS) visible in all echocardiographic views (Fig. 1A, B, C). There were no signs of other cardiac lesions such as valvular heart disease or atrial septal defect. Pulsed-wave doppler did not reveal any abnormal shunting between the left atrium and the CS. As there was a suspicion of PLSVC hand-agitated saline was injected via peripheral intravenous cannulas. The contrast appeared firstly in the huge CS before it opacified the right atrium (Fig. 1D, E; Suppl. Video). Since this was also visible by the right antecubital saline injection, it indicated a rare case of PLSVC with the absence of right SVC. The diagnosis was confirmed by cardiac magnetic resonance imaging which visualized the isolated PLSVC using standard steady-state free precession acquisitions, covering the entire chest in orthogonal (i.e. in both transverse and coronal) 8 mm thick cross-sections with 2 mm spacing to delineate anatomy. These acquisitions pertained to a standard cardiac protocol, namely to avoid missing rare but potentially important extracardiac findings (Fig. 1F, G, H).

## Discussion and conclusions

Vascular anomalies represent a broad spectrum of different pathologies involving arteries, veins and lymph vessels [2–5]. Although taking into account all of them, PLSVC is infrequent, it is the most common congenital malformation of thoracic venous return that affects 0.2 to 3% of the healthy population [6]. On the other hand, PLSVC with absent right SVC is an extremely rare venous anomaly [2]. PLSVC forms when the left anterior cardinal vein fails to obliterate during fetal life [1]. It usually originates from the junction of the left subclavian and internal jugular veins, proceeds through the left side of the mediastinum adjacent to the aortic arch. It mostly connects to the right atrium via the CS widening its structure. Less frequently PLSVC drains into the left atrium directly or through an unroofed CS which leads to right-to-left shunt. In current case, the humongous CS (up to 29 mm in apical two- chamber view, with normal values set at 8.27 ± 2.5 mm [7]) might suggest that it was a vascular variant of only one SVC present. The isolated PLSVC carries the entire upper body venous return leading to a huge size of the CS, larger than in the co-existence of the right-sided SVC simultaneously draining directly into the right atrium. Moreover, the fact that both left and right antecubital contrast injection reached the same result, that is, opacification of the enormous CS prior to right atrial filling, indicated the diagnosis of isolated PLSVC. In most cases it causes no hemodynamic consequences and is usually discovered incidentally on either imaging or during intervention. Nevertheless, the proper in-depth diagnosis is vital. PLVSC is a potential factor triggering common arrhythmia- atrial fibrillation, and the presence of isolated PLVSC increases the risk of complications during left atrial ablations [8]. More frequent monitoring for atrial fibrillation and special preparation in case of indications for ablation may be relevant. PLSVC may also have other important clinical implications, such as difficulties with central venous access, cardiothoracic surgeries, and pacemaker or defibrillator implantations, especially in the current era of a rapid development of cardiovascular device therapies with reference to increasing life expectancy and ageing of population.

### Electronic supplementary material

Below is the link to the electronic supplementary material.

**Supplementary Video**: Bubble contrast echocardiography. Hand-agitated saline was injected via peripheral intravenous cannulas. The contrast appeared firstly in the huge coronary sinus before it opacified the right heart. As the same situation appeared by left and right antecubital saline injection, it indicated a rare case of isolated PLSVC. PLSVC- persistent left superior vena cava

## Data Availability

Records and data regarding this case are in the patient’s secure medical records in the Department of Cardiac Diagnostic, Gdansk, Poland.

## Abbreviations

PLSVC: persistent left superior vena cava
SVC: superior vena cava
CS: coronary sinus
